# An epidemiological qualitative/quantitative SWOT‐AHP analysis in order to highlight the positive or critical aspects of dental implants: A pilot study

**DOI:** 10.1002/cre2.836

**Published:** 2024-03-07

**Authors:** Silvana Mirella Aliberti, Richard H. W. Funk, Marco De Stefano, Thomas Hoffmann, Mario Capunzo

**Affiliations:** ^1^ Department of Medicine, Surgery and Dentistry, “Scuola Medica Salernitana” University of Salerno Salerno Italy; ^2^ Institute of Anatomy Technische Universität (TU) Dresden Dresden Germany; ^3^ Division of Preventive Medicine Dresden International University (DIU) Dresden Germany; ^4^ Department of Industrial Engineering University of Salerno Salerno Italy

**Keywords:** aging, dental implants, disease‐biomechanical problem, SWOT‐AHP analysis

## Abstract

**Objectives:**

In recent years, dental implants are increasing in popularity due to their high success rate, demonstrated functionality, and aesthetic treatment results. Scientific research is very active in proposing improvements in the quality and survival of implants, taking into consideration various aspects. The objective of this study was to provide a holistic epidemiologic view of the state of dental implants, using a systematic approach based on a multimethod SWOT (Strengths, Weaknesses, Opportunities, and Threats) analysis and AHP (analytical hierarchical process) qualitative–quantitative analysis to identify the characteristics that can determine their success or failure.

**Materials and Methods:**

The study used the hybrid method of SWOT‐AHP.

**Results:**

Analysis of the results showed that among strengths, the skill of the dentist was considered the most important factor, followed by the success of dental implants in the old people; among weaknesses, bruxism and chronic diseases were highlighted; for opportunities, biomechanical behavior, in terms of good mechanical strength and good tribological resistance to chemical and physical agents in the oral cavity, were considered the most important factors; finally, among threats, medical liability and biomechanical problems had equal weight.

**Conclusions:**

This study applied a multimethod SWOT‐AHP approach to bring out favorable or critical evidence on the topic of dental implants. In accordance with the result of the strategic vector identified in the Twisting zone Adjustment type section, showed that implant surgery is a widespread technique but always needs improvement to increase the likelihood of success and reduce the complications that can lead to implant failure.

## Abstract (highlights)

**What is known?:**

- Many factors like aging, common diseases, and biomechanical issues early account for the implant success.
- Sinus floor elevation combined with conventional length implants results in increased cost and treatment time, risk of infection, postoperative sinusitis, limited bone gain.
- Shorter implants can result in increased biomechanical complications, including loosening and breakage of the implant screw and prosthesis, bone loss.
- Both procedures include short‐term failures due to overheating of the bone during preparation of the implant site, lack of primary stability due to over‐preparation or poor bone quality, overloading, and others.
- Bone loss due to peri‐implantitis or implant fractures are the main causes of long‐term implant failure.
- General implant survival is influenced by tobacco smoking, periodontal disease, systemic disease, bone atrophy, and related factors.

**What this study adds?:**

Because the interplay or more parameters can lead to lower implant success, we introduce here a

- novel calculation method for these factors to provide a holistic epidemiological view of the status of dental implants, adopting a systematic approach based on:
- a multimethod qualitative–quantitative analysis of strengths, weaknesses, opportunities, and threats (SWOT) and
- analytical hierarchical process (AHP) to bring out the favorable or critical aspects and define a possible strategy for action.

## 1 INTRODUCTION

According to the World Health Organization (WHO), oral health enables individuals to perform essential functions and increases well‐being, self‐esteem, the ability to socialize without discomfort, and to realize one's work potential (WHO, 2022). On the other hand, dental caries, periodontitis, and traumatic events are the major causes of tooth loss. Worldwide, the prevalence of (partially) edentulous patients ranges from 7% to 69% (Petersen et al., 2005). Fixed and removable prostheses have been used to restore chewing function and esthetics, while dental implants have been a valuable adjunct to replace damaged or missing teeth and provide oral health rehabilitation (Solderer et al., 2019). In fact, dental implants have become increasingly popular in recent years due to improved functional and esthetic treatment outcomes (Hjalmarsson et al., 2016; Wittneben et al., 2014). According to Torabinejad et al. (2007), the success rate of implant rehabilitation is 95%, while the success rate of fixed dentures on teeth is 81%. However, it should be noted that both the atrophic edentulous maxilla and mandible undergo a morphologic remodeling of the bone tissue by reducing the alveolar ridge in horizontal and vertical dimensions (Iezzi et al., 2020). To compensate for the bone loss, the dentist must decide which technique to use. The option, considered the gold standard (Thoma et al., 2017), is the use of sinus floor elevation in combination with conventional length implants. With this technique, the survival rate of implants in long‐term evaluations is more than 90%.

This invasive procedure has many disadvantages, such as increased cost and treatment time, risk of infection, postoperative sinusitis, limited bone gain, and, in addition, good outcome depends on the skill of the operator (Iezzi et al., 2020). The other option is to use shorter‐length implants to overcome the problem of bone deficiency (Schiegnitz et al., 2022). By choosing short implants and avoiding augmentation surgery, treatment time, cost, and patient morbidity are reduced, resulting in greater patient satisfaction and compliance (Nielsen et al., 2021; Schiegnitz et al., 2017; Svezia & Casotto, 2018; Tang et al., 2020).

Both implant and prosthetic failures and complications are frequently observed over time (Bozini et al., 2011; McGlumphy et al., 2019). Biological, biochemical, and biomechanical complications, including loosening and fracture of the implant screw and prosthesis, bone loss and the possibility of infection, can compromise the success of the implants (Brägger et al., 2001; Chatzopoulosa & Wolffa, 2023; El Askary et al., 1999). According to some prospective studies, technical variables seem to have a greater influence than biological variables on the complications of implant‐supported complete fixed prostheses (Coltro et al., 2018; Pauletto et al., 2019), mainly because it is less likely to face biological issues. Several independent factors have been proposed in the literature that may influence implant survival. These may include tobacco smoking, periodontal disease, systemic disease, significant bone loss and poor bone quality, proper placement of the implant in the arch and fresh extraction sites, use of long inferiors, lack of implant stability, and the need for experienced surgeons with several years of experience to perform the procedure (Chrcanovic et al. 2014, 2016). Of course, when more parameters are evaluated, implant success rates are lower (Papaspyridakos et al., 2012). Implant failure can be characterized as an early or late event. While early failures occur at an early stage due to inadequate osseointegration, that is, before the implants are used and functionally loaded, late implant failure, after osseointegration has occurred, are usually associated with reduced bone support (Esposito et al., 1998). Causes of early implant failure may include overheating of the bone during implant site preparation, lack of primary stability due to overpreparation of the implant site or poor bone quality, overloading, or parafunctions (Froum et al., 2011). At this stage, implants are easy to remove because they are clinically mobile. In contrast, late implant failures are mainly due to biological or biomechanical reasons, such as bone loss due to peri‐implantitis (De Stefano et al., 2022) or implant fractures. Experimental investigations and observations of fractured implants show that a nonadequate mechanical surface treatment can generate surface's microfractures which reduce the mechanical resistance leading the implant fracture (Monzavi et al., 2020; Scherrer et al., 2019).

It is important to consider the cause of implant loss to prevent future implant failures. Implant failure has a significant implication for both patient and dentist, so identifying implant failure risk factors can help clinicians plan treatment and promote a long‐term success.

The main objective of this study was to provide a holistic epidemiological view of the state of dental implants, adopting a systematic approach based on a multimethod qualitative–quantitative analysis, SWOT (Strengths, Weaknesses, Opportunities, and Threats) and AHP (Analytical Hierarchical Process) to identify the positive or critical aspects that determine their success or failure. More in detail, the SWOT elements were achieved by a group of experts including dentists and engineers who provided the basis for the construction of AHP model following Saaty nine‐point scale criterion for the factors weight. Finally, the results were analyzed and discussed in depth.

Although there are many studies on dental implants, to the best of our knowledge, this is one of the first studies to use a multiple methodological approach to provide positive or critical evidence on the topic.

## 2 MATERIALS AND METHODS

### 2.1 Study design and sampling

The study was conducted using a hybrid SWOT‐AHP model between September 2022 and March 2023 on a sample of dental implant experts. Avalanche sampling was used to recruit the sample, initially involving a few dentists who were explained the purpose and method of the study and asked to involve other dentist or implantologist colleagues. At the end of the recruitment period, the sample consisted of a total of 18 experts, including three implantologists, 13 dentists, and two mechanical engineers (experts in biomechanics and biotribology).

### 2.2 Data collection instrument

The study was based on an open‐ended questionnaire administered to stakeholders as input for the focus group and on the proposal of a hybrid approach based on the SWOT‐AHP model analysis methodology. The SWOT analysis is a technique used to identify the SWOT related to a specific project. In our case, the SWOT model was used to classify the favorable or critical factors underlying dental implants through a matrix in which intrinsic (strengths and weaknesses) and extrinsic (opportunities and threats) elements are correlated. Strengths are those factors that can produce good results, while weaknesses are those factors that can draw a line between success and failure. Opportunities are those factors that could improve weaknesses, while threats are those factors that could cause problems (they differ from weaknesses in that they are generally out of our control). In turn, the AHP model, which is a multicriteria decision analysis method, was used to assign importance to each factor included in the SWOT matrix and to distinguish the weight of each factor (Chang & Huang, 2006; Dos Santos et al., 2019; Helms & Nixon, 2010; Subramanian & Ramanathan, 2012). This hybrid method has been successfully applied in scientific research fields (Brudermann et al., 2015; Hao et al., 2022; Kurttila et al., 2000; Li et al., 2021; Reinsberger et al., 2015).

Therefore, the study design was conducted in three main steps: (1) individuation of potential SWOTs related to dental implants through stakeholders; (2) quantification of the most relevant SWOT points (AHP method) by the same stakeholders; (3) finally, the results obtained were analyzed and discussed within the recent and pertinent scientific literature, to select the most relevant research directions in the field.

### 2.3 Generating the factors included in the SWOT analysis

**Figure 1:**
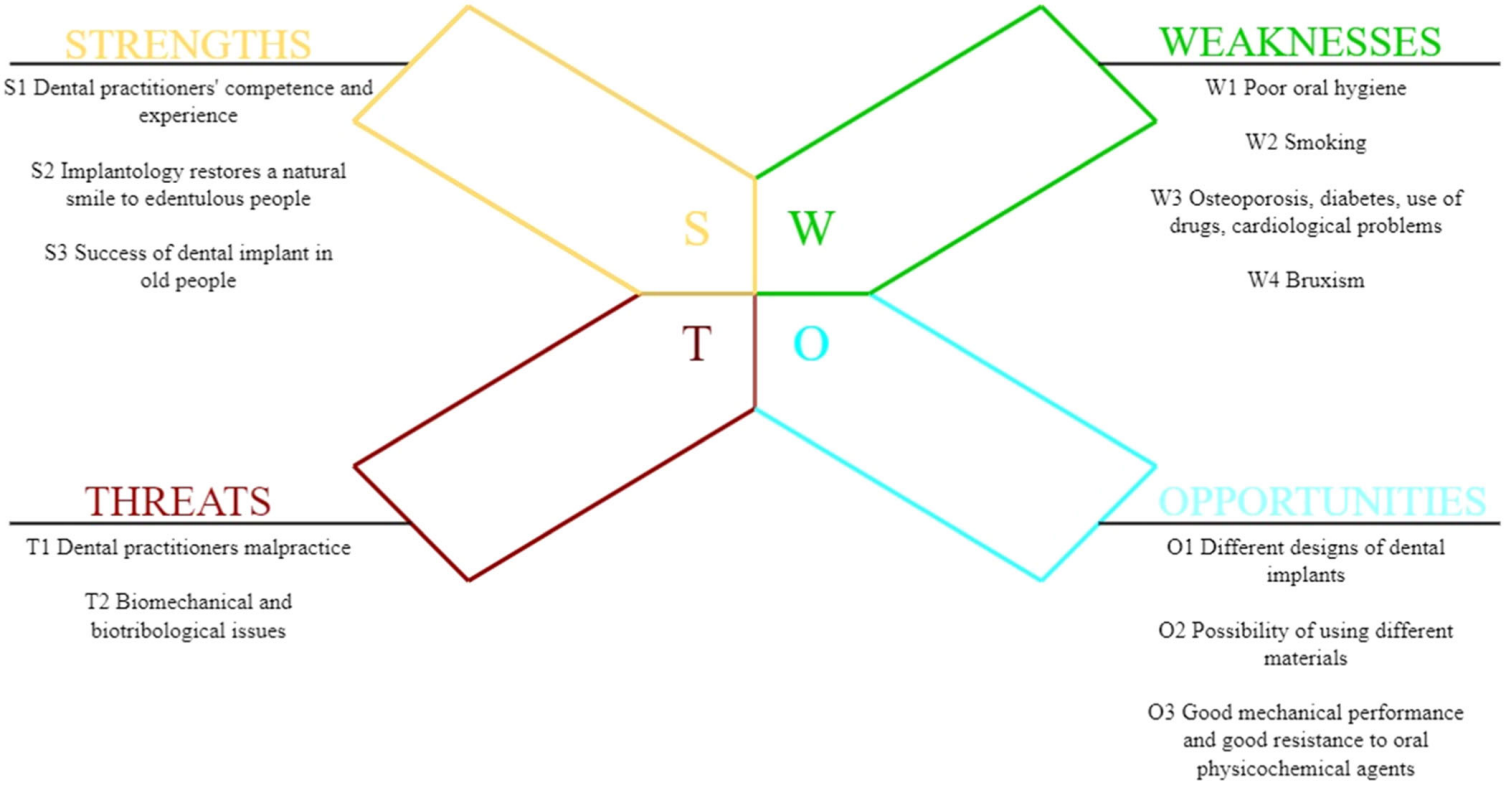
The stakeholders' responses were systematized according to the Strengths, Weaknesses, Opportunities, and Threats method.

The SWOT factors were selected based on three focus groups organized with experts (three implantologists, 13 dentists, and two mechanical engineers), all with 20 or more years of experience, on implantology issues. Following a guideline of open‐ended questions (what aspects should be considered before implant placement, is advanced age an issue, what are the advantages or disadvantages of dental implants), the topic was discussed extensively to ensure that the proposed variables and their respective categories were relevant to implantology. Discussions were held with 18 people to allow for brainstorming and to avoid the prevalence of individual perspectives. Based on the SWOT matrix, the experts selected 10 factors that were grouped into each SWOT category (Figure 1).

### 2.4 Ethical approval

The tenets of the Declaration of Helsinki guided the design and conduct of the study. The protocol fully assured anonymity and confidentiality of the participants and the absence of risk, conflict of interest, or inducement to the participants. Importantly, no patients or clinical data were used in this study, and the results are based on the opinions of dental experts (implantologists, dentists, and mechanical engineers) who provided formal informed consent.

### 2.5 AHP models

The second step of the method application involved pairwise comparisons and the weighting of the SWOT factors, through the AHP hierarchy. Based on the discussions, experts in the field were tasked with assigning weights and ratings to the identified SWOT factors. The weights were assigned considering the impact of the weaknesses and threats versus the positive impact of the selected strengths and opportunities. A nine‐point scale proposed by Saaty (2008) was applied to each identified factor, ranging from 9/1 (i.e., factor a is much more important than factor b) to 1/9 (i.e., factor a is much less important than factor b). Even numbers indicate intermediate levels. The middle part of the scale (1/1) indicates that the respective factors are equally important (Table 1).

**Table 1:** Saaty's Scale of relative importance.

Intensity of importance	Definition	Explanation
1	Equal importance	Two activities contribute equally to the objective
2	Equally to moderately	Importance between the above levels
3	Moderate importance	Experience and judgment slightly favor one activity over another
4	Moderately to strongly	Importance between the above levels
5	Strong importance	Experience and judgment strongly favor one activity over another
6	Strongly to very strongly	Importance between the above levels
7	Very strong or demonstrated importance	An activity is favored very strongly over another; its dominance is demonstrated in practice
8	Very strong to extremely	Importance between the above levels
9	Extreme importance	The evidence favoring one activity over another is of the highest possible order of affirmation
1/2, 1/3, 1/4, 1/5, 1/6, 1/7, 1/8, 1/9	If subfactor A is less important than subfactor B, then intensity of importance of A is the inverse of the intensity of importance of B	A reasonable assumption

### 2.6 Calculation of subfactor weights and consistency ratio (CR)

Since people tend to have different judgments and preferences, the scores assigned to the two subfactors to be compared were not always equal, so a mean of the experts' responses was calculated after two rounds of information. In addition, the CR, which is a measure for establishing the validity of respondents' answers, was used to assess their consistency. STATA software (STATA, 2019) was used for statistical calculations.

The methods and calculation steps follow (Liu et al., 2019; Saaty, 2008; Wang & Chen, 2010):

1. Construct comparison matrix A (Equation 1.1).
(1.1) $$Α=\left(aij\right)=\left[\begin{array}{lll} 1 & w_{1}/w_{2} & \cdots \;w_{1}/w_{n}\\ w_{1}/w_{2} & 1 & \cdots \;w_{2}/w_{n}\\ w_{n}/w_{1} & w_{n}/w_{2} & \cdots \;1 \end{array}\right].$$
2. Calculate the consistency index (CI) and the CR (Equation 1.2).

(1.2) $$\mathrm{CI}=\frac{\lambda_{\max-n}}{n-1}.$$

To test the CI adequacy, the CR, defined by the ratio of the CI to the random consistency index (RI), was calculated. The matrix is considered consistent if the CR < 0.1, while the matrix is failed if CR > 0.1 (Equation 1.3).

(1.3) $$\mathrm{CR}=\frac{\mathrm{CI}}{\mathrm{RI}}.$$

The RI values are shown in Table 2.

**Table 2:** RI.

N	1	2	3	4	5	6	7	8	9	10
RI	0	0	0.58	0.90	1.12	1.24	1.32	1.41	1.45	1.49

Abbreviation: RI, random consistency index.

### 2.7 Calculation of the SWOT factor intensity and construction of the SWOT strategy quadrilateral

The effect size of the factor is given by the estimated power time its weight. The estimated power of each factor is represented by 0–5 points. S (Strengths) and O (Opportunities) are represented by positive values, while W (Weaknesses) and T (Threats) represented by negative values. They form a coordinate system with four semidimensions. To obtain a strategic quadrilateral, the absolute values of S, W, O, T must be plotted in the corresponding semiaxes of the coordinate system.

### 2.8 Calculation of strategic vector (θ,ρ)

**Figure 2:**
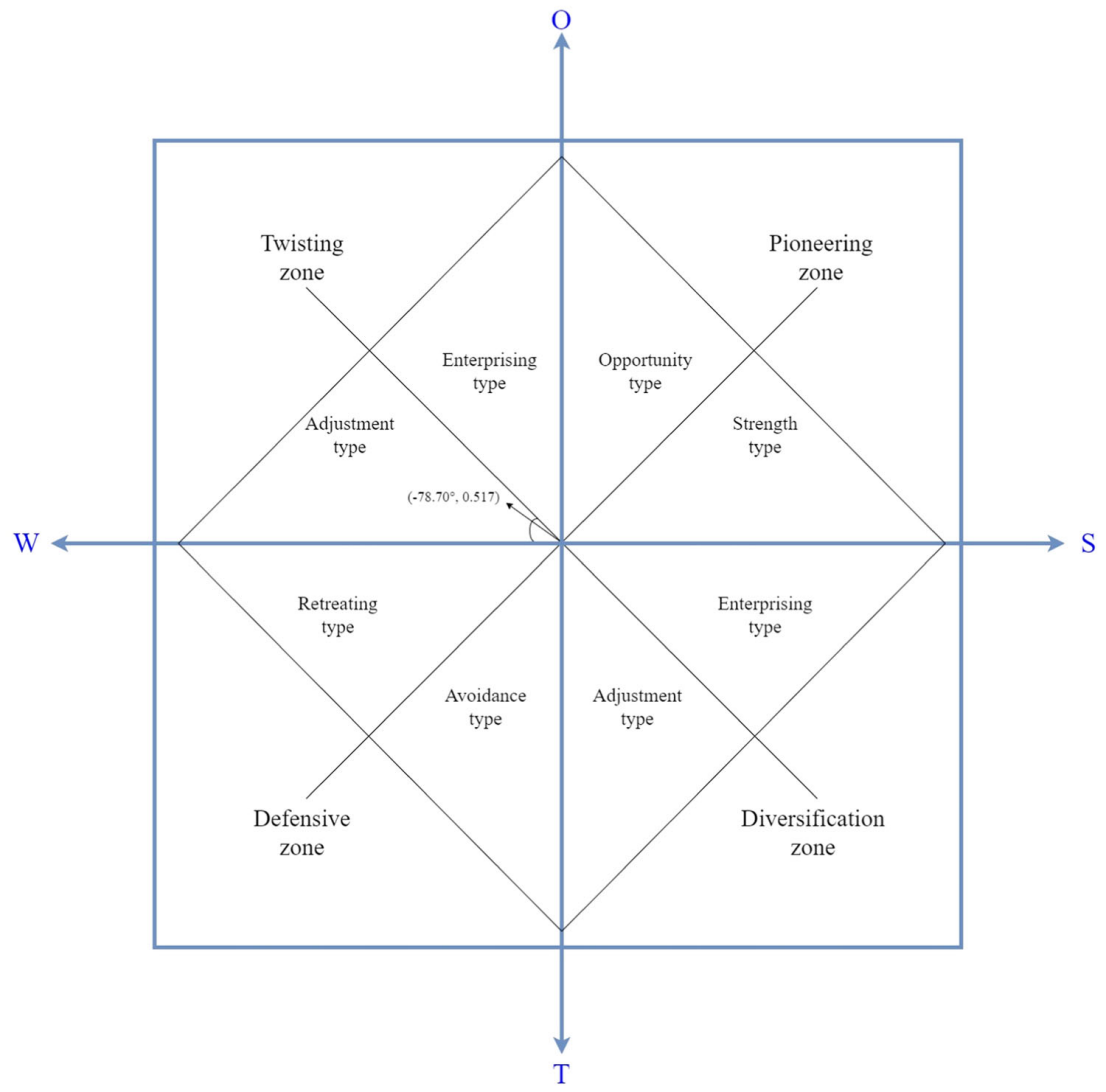
Strengths, Weaknesses, Opportunities, and Threats strategic vector and strategic quadrilateral. The strategic quadrilateral ∑ Oi = 4.370 > ∑ Wi = −4.243 > ∑ Si = 4.169 > ∑ Ti = −4.000. The coordinates of the center of the strategic quadrilateral are P (X, Y) = (−0.0185, 0.0925). The strategic azimuth is *θ* = arctan $\left(\frac{0.0925}{-0.0185}\right)$ ≈ −78.70°. The strategic positive intensity is U = 18.218. The strategic negative intensity is V = 16.972. The strategic strength coefficient is ρ = 0.517.

The strategic azimuth angle *θ* and the strategic intensity coefficient ρ are used to define the strategic type and strategic intensity, respectively, in the SWOT‐AHP model (For a better understanding, have a look at the calculation in the note to Figure 2).

The strategic azimuth is (Equation 1.4):

(1.4) $$\theta =\arctan\;\frac{Y}{X}.$$

1. Calculation of the coefficient of strategic intensity ρ, which can be positive (U) or negative (V). Positive strategic intensity is the result of the joint action of intrinsic strengths and extrinsic opportunities (Equation 1.5).

(1.5) U=O'×S'.

Negative strategic intensity is the result of the combination of intrinsic weaknesses and extrinsic threats (Equation 1.6).

(1.6) V=T'×W'.

The strategic intensity factor ρ is calculated as follows (Equation 1.7):

(1.7) $$\rho =\frac{U}{U+V}.$$

## 3 RESULTS

### 3.1 AHP weights, SWOT strategic quadrilateral, and strategic vector

The results of the weighting assigned by the experts to the negative impacts of weaknesses and threats and the positive impacts of strengths and opportunities of the identified SWOT factors showed that opportunities have the most important consideration, followed by weaknesses, strengths, and threats. In the category of strengths, the skill of the dentist was considered the most important factor, followed by the success of dental implants in the old and implantology restoring a natural smile to edentulous people. Among weaknesses, bruxism and chronic diseases were considered the most important factors, followed by poor oral hygiene and smoking. Among opportunities, biomechanical behavior, in terms of good mechanical strength and good tribological resistance to chemical and physical agents in the oral cavity were considered the factors with the most weight, followed by the possibility of using different materials and different designs of dental implants. In terms of threats, dentist malpractice and biomechanical and biotribological problems had equal weight. The weights and intensities of the factors are summarized in Tables 3 and 4. The strategic quadrilateral, which shows the results of the calculation of the total intensities of each group (∑ Oi = 4.370 > ∑ Wi = −4.243 > ∑ Si = 4.169 > ∑ Ti = −4.000) was plotted on the abscissa and ordinate axes of the strategy diagram (Figure 2). The diagram shows that the strategic vector is in the Twisting zone, particularly in the section of the Adjustment type that tends to the Enterprising type (coordinates θ,ρ = −78.70, 0.517) (Figure 2), indicating that optimal adjustment is needed for dental implants to show intrinsic benefits.

**Table 3:** Comparison of SWOT groups, matrices, and factor weights.

SWOT group	Comparison matrix	Factor weight	Maximum eigenvalue	CI	CR
Strengths (S)	$\left[\begin{array}{lll} 1 & 5 & 3\\ 1/2 & 1 & 1/3\\ 1/3 & 3 & 1 \end{array}\right]$	WS1 = 0.637 WS2 = 0.105 WS3 = 0.258	3.039	0.019	0.033
Weaknesses (W)	$\left[\begin{array}{llll} 1 & 5 & 5 & 1\\ 1/5 & 1 & 1/5 & 1/7\\ 1/5 & 1 & 1 & 1/5\\ 1 & 7 & 5 & 1 \end{array}\right]$	WW1 = 0.179 WW2 = 0.045 WW3 = 0.219 WW4 = 0.557	4.121	0.040	0.044
Opportunities (O)	$\left[\begin{array}{lll} 1 & 1/5 & 1/7\\ 5 & 1 & 1/3\\ 7 & 3 & 1 \end{array}\right]$	WO1 = 0.072 WO2 = 0.279 WO3 = 0.649	3.065	0.032	0.068
Threats (T)	$\left[\begin{array}{ll} 1 & 2\\ 1/2 & 1 \end{array}\right]$	WT1 = 0.500 WT2 = 0.500	2.000	0.001	0.001

*Note*: CR < 0.1 pass the consistency check.

Abbreviations: CI, consistency index; CR, consistency ratio; SWOT, Strengths, Weaknesses, Opportunities, and Threats.

**Table 4:** Groups and factors intensity.

SWOT group	Factor weight	Estimated strength	Factor intensity	Total intensity
Strengths (S)	W_S1_ = 0.637 W_S2_ = 0.105 W_S3_ = 0.258	5 2 3	3.185 0.210 0.774	∑ Si = 4.169
Weaknesses (W)	W_W1_ = 0.179 W_W2_ = 0.045 W_W3_ = 0.219 W_W4_ = 0.557	−3 −1 −4 −5	−0.537 −0.045 −0.876 −2.785	∑ Wi = −4.243
Opportunities (O)	W_O1_ = 0.072 W_O2_ = 0.279 W_O3_ = 0.649	1 3 5	0.072 1.053 3.245	∑ Oi = 4.370
Threats (T)	W_T1_ = 0.500 W_T2_ = 0.500	−4 −4	−2.000 −2.000	∑ Ti = −4.000

Abbreviation: SWOT, Strengths, Weaknesses, Opportunities, and Threats.

Table 4 shows the strategic strength of each subfactor and the total strength of the SWOT‐AHP model.

## 4 DISCUSSION

The objective of this study was to provide a perspective on dental implants, with the aim of identifying the most relevant research directions in dental implantology, using a systematic approach based on a qualitative–quantitative, multimethod SWOT‐AHP analysis. In view of the results obtained, several interesting issues emerged.

The diagram shows that the strategic vector is in the area of Twisting, particularly in the section of the type Adjustment tending to the type Enterprising, which means that implant surgery is a popular technique but that further improvements are needed to increase the probability of success and reduce complications. The potential problems of dental implants can be both biological and mechanical in origin, according to the results of our study (SWOT matrix related to threats). From a clinical point of view, incomplete osseointegration can lead to prosthesis failure. On the other hand, from a mechanical point of view, fracture or functional loosening is also crucial. The fixation screw could be a weak point in the system, according to Cervino et al. (2018), in fact, the stresses transmitted to it are often higher than not only the yield stress but also the ultimate strength, leading to failure of the component and thus the whole system. The above causes are not exclusive and can also act simultaneously, strongly affecting the mechanical properties of the system. The synergistic action of corrosion and mechanical wear is known as tribocorrosion (De Stefano et al., 2022) and has a significant impact on the survival of the prosthesis. To activate intervention strategies, according to the results of the SWOT matrices, the first approach (opportunity subfactors, found to be the most important requirement) is to aim for the activation of good mechanical performance and good resistance to oral physicochemical agents of dental implants. The material used in dental implants must possess specific physical, mechanical, and chemical and biocompatibility properties, such as mechanical toughness and hardness, as well as resistance to corrosion and tribo‐corrosion caused by continuous exposure to external weathering. For this reason, implants can be treated on their microstructure (Sevilla et al., 2010) and/or with appropriate surface treatments that act on roughness, wettability, hardness, and so on (Jiang et al., 2020) with the aim of increasing their mechanical and biotribological performance. Appropriate coatings can also be used to promote the formation of a hard and stable oxide layer that prevents microdissolution by external agents (Nagay et al., 2022). In agreement with Wiskott and Belser (1999), who have evaluated the effect of roughness, they state that a rough tread surface is preferable because it generates a stress regime that can promote bone formation and promote osseointegration, that is, bone growth near the implant. Regarding the choice of implant material, it is almost obvious that an optimal type cannot be guaranteed because several characteristics are involved. The most widely used are commercially pure titanium or grade V titanium because of their remarkable biocompatibility and Young's modulus very close to that of bone, thus reducing potential bone resorption. However, the possibility of ion release from these materials is a major drawback because it can cause inflammation or more dangerous problems (Affatato et al., 2018). Therefore, in 2015, Elias et al. (2015) proposed grade 4 hard titanium as an alternative without compromising the fracture toughness and mechanical properties of the implant. Other options, such as polymers, do not exhibit the same mechanical behavior, as demonstrated by Sarot et al. (2010) in the contrast between titanium implants and CFR‐PEEK (carbon fiber reinforced polyether ketone). On the other hand, a more elastic material can absorb and dissipate stresses faster than metal, thus avoiding potential bone resorption (Cantó‐Navés et al., 2021). Zirconia, on the other hand, has generated mechanical performance comparable to that of the titanium implant for both stresses and displacements and thus could be adopted (Chang et al., 2012).

Regarding the mechanical aspect, many authors have studied specific geometric characteristics, such as the type of threading and its impact on bone stress, in agreement with Zarei et al. (2016), that is, the abutment, which is fundamental as demonstrated by Cicciù et al. (2019), especially in terms of marginal bone loss (Del Amo et al., 2022) and diameter size (Raikar et al., 2017), which is highly variable in the literature (Al‐Johany et al., 2017). In this scientific framework, there is a new trend toward short or ultrashort implants market, <8 mm and 6 mm in length, respectively. The advantages are almost obvious, such as reduced postmandibular height (Annibali et al., 2012), but also less invasive than long ones, especially considering the location of the inferior alveolar nerve (Lemos et al., 2016), with less likelihood of inflammation and faster recovery time. In addition, as demonstrated by De Stefano et al. (2023), comparison between a newly designed implant with a thick shape, length <5 mm and diameter‐to‐length ratio >1, and a classically narrow shape implant with a length >5 mm and diameter‐to‐length ratio <1, showed better mechanical performance for the short ones. In fact, the distribution of implant stresses and strains was more uniform with lower peaks for all boundary conditions considered. On the other hand, it should be noted that the reduced contact area with the bone may compromise osseointegration and subsequent stability. Therefore, increasing the implant diameter or design area could be helpful to overcome this problem (Misch, 2005). Recently, analog root implants are capturing the attention of clinicians due to their low invasive properties, improved bone stress distribution, and the not requirement of bone drilling, sinus elevation, and other traumatic procedures (Dantas et al., 2020).

As mentioned above, there are several implant alternatives for people suffering from edentulism. However, the patient's pathological history is of considerable importance. The AHP results show among the weaknesses (second most important factor), that pathologies such as bruxism and chronic diseases were considered important factors that may play in favor of dental implant success or failure, followed by poor oral hygiene and smoking. Bruxism, one of the potential risk factors for dental implant failure (first among the subfactors of weaknesses), is a movement disorder of the masticatory system as repetitive activity of the jaw muscles whose most prominent features are clenching or grinding of the teeth and tilting or thrusting of the jaw. This etiologic factor damages the supporting structures of teeth, causes temporomandibular disorders, musculoskeletal pain, failure of dental restorations, and tooth wear (Behr et al., 2012; Lobbezoo et al., 2013; Zhou et al., 2016). The increased risk of implant failure is due to uncontrolled functional loading, which could produce micromovements preventing osseointegration by embedding the implant in a fibrous tissue (Dutta et al., 2020). Bruxism is usually considered a contraindication to dental implantation, which is based only on the dentist's experience (Grandi et al., 2012). This is in agreement with Zhou et al. (2016) who showed significant association between bruxism and implant failure in their meta‐analysis study. Implants in bruxists have a higher failure rate than in nonbruxists. The most common complications are chipping or fracture of porcelain on implant‐supported crowns.

Another risk factor for dental implants is osteoporosis, which causes bone weakness and fragility, and a reduction in the mineral density (mass/volume units) of bone (Berry & Nedivi, 2017). The mandible and maxilla share the same altered bone metabolism as other bones in the body (Mori et al., 1997), so the concern is also related to the idea that implant osseointegration may be affected by decreased bone metabolism related to osteoporosis (Sendyk et al., 2017).

Implant failure may also be caused by certain medications. Corticosteroids are commonly used to treat various systemic diseases. Patients using exogenous steroids may develop osteopenia and osteoporosis and are more likely to experience a decrease in bone density. Systemic users are more likely to experience decreased bone density, increased epithelial fragility, and immunologic suppression, which affect dental implant osseointegration (Kochar et al., 2022).

Patients with type 2 diabetes may be at increased risk for postoperative complications due to infection or slow wound healing. In addition, patients with diabetes are three times more likely to develop periodontitis (Aljofi et al., 2023; Preshaw & Bissett, 2013). Antidiabetic drugs such as metformin have been shown to have a beneficial effect on bone tissue, acting on osteoblasts and reducing the risk of fractures in patients with diabetes (Bak et al., 2010; Cortizo et al., 2006). To avoid problems, the dentist should take the patient's history, including considering previous HbA1c levels, to determine how well or poorly the patient is controlled (Mealey, 2014). Because diabetic patients with inadequate glycemic control are most at risk for postoperative surgical complications, it is necessary to achieve adequate glycemic control before dental implant surgery.

After a myocardial infarction or cerebrovascular accident, the dentist should wait until the patient is stabilized because of the high risk of complications. The patient may undergo elective dental treatment only if at least 6 months have passed since the ischemic event and medical clearance has been obtained (Hwang & Wang, 2006). The dentist should be aware of any anticoagulant or thrombolytic therapy being administered and understand that the placement of oral implants does not always warrant discontinuation of such treatments (Niwa et al., 2000).

Infection is the most frequent and avoidable cause of dental implant failure. Poor oral hygiene, microscopic spaces between implant components, and retained cements in the subgingival area induce bacterial colonization that causes peri‐implantitis (Kochar et al., 2022). Peri‐implantitis is a local inflammatory response with bone loss in the soft tissues surrounding the implants (Boccia et al., 2023) that can lead to implant failure. Patients should be thoroughly educated on proper oral hygiene techniques, paying particular attention to cleaning implant sites. Therefore, further investigations might focus more on preimplant diseases and their prevention and treatment (Herrera et al., 2023).

Smoking is a global risk factor affecting 1.4 billion people worldwide and is associated with numerous oral diseases, including cancer, cardiovascular disease, and periodontal disease (Leite et al., 2018; Reitsma et al., 2021; West, 2017). In addition, smoking can contribute to poor healing and reduced blood supply, resulting in increased marginal bone loss and dental implant failure rates (Chrcanovic et al., 2016). In fact, in the survival rate of dental implants is reduced in smokers (Kochar et al., 2022). This agrees with research by Mustapha et al. (2021), who found that smoking patients had a 140.2% higher risk of dental implant failure and more significant marginal bone loss than nonsmokers. According to Meyle et al. (2019), smokers have a 2.25 times greater risk of losing a functionally loaded implant than nonsmokers after implant placement in native bone. This risk increases to 3.61 when implants are placed in augmented bone. Smokers with poor oral hygiene have a higher risk of progressive bone loss than nonsmokers with similar levels of oral hygiene. The dentist's goals must be multifaceted for each edentulous patient who is a candidate for implant placement: achieving an ideal esthetic outcome is one of the most important goals of implant‐prosthetic therapy, but improving oral health and establishing proper occlusal function are also necessary goals for a successful outcome (Jivraj & Reshad, 2018).

On the other hand, in agreement with our AHP findings among the strength categories, one of the predictors of dental implant success is the dentist's adequate expertise and experience in diagnosing, planning, and implementing of implant‐prosthetic rehabilitation, as well as the patient education in the self‐care. For example, improper oral hygiene during the life cycle of the prothesis may contribute to the development of clinical complications. According to Sendyk et al. (2017), who performed a meta‐analysis to evaluate the effect of dentist expertise on implant failure, surgical experience does not significantly affect implant failure when considering experience by specialty (odds ratio [OR] = 1.24; 95% confidence interval [CI], 0.62–2.48; *p* = .54), but it does significantly affect implant failure when considering experience by number of implants placed (OR = 2.18; 95% CI, 1.40–3.39; *p* = .005). The results of the study by Rozov et al. (2019), which was aimed at developing a system to assess the actual skill level of implantologists, are consistent with the findings that dentists who have been practicing implantology for 1–7 years have good manual skills and sufficient clinical competence to achieve successful treatment results. At the same time, their actual diagnostic skills are generally inadequate in terms of correct clinical description of patient oral cavity, they misapply additional diagnostic methods, and they do not perform risk analysis of potential clinical complications, which results in shifting the responsibility to the patients in case of complications. Computer‐aided design methods are widely used in all phases of rehabilitation (diagnostic, surgical stent, final prosthetic structure). It is an accurate and robust method based on engineering optimization of the entire workflow. Starting with the scanning of the patient's oral cavity and the construction of a 3D model, it involves the use of surgical guides, made with specific treatments such as stereolithography (Gallardo et al., 2016). In this way, dental implants are placed more efficiently and accurately (Lin et al., 2015), with results relevant to proper implant restoration (Rungcharassaeng et al., 2015) and minimizing the risk jawbone damage (Casap et al., 2004).

Another aspect that emerges from the AHP results of our study is that implant treatment in the old is comparable to that observed in young adults (Bryant & Zarb, 1998; Engfors et al., 2004). Several previous studies have shown that due to their medical conditions and the adverse effects of medications taken, old patients undergoing implant surgery are more at risk than younger patients (Akkus et al., 2004; Wang et al., 2002). Current studies, on the other hand, have shown that the old population has increased life expectancy not only in terms of duration but above all in the increase in the healthy part of life (Aliberti, De Caro, et al., 2022; Aliberti, Funk, et al., 2022; Aliberti et al., 2023). For similar reasons, implant treatment for old patients is on the rise worldwide (Choi et al., 2023). In agreement with Köndell et al. (1988), who compared the success rate of 284 implants in 53 old patients, aged 65–85 years with the success rate of 183 implants in 36 younger patients, aged 18–54 years. The implants supported mostly partial or complete fixed prostheses and some overdentures. The overall success rate, after 1–6 years of follow‐up was the same or slightly better in the older patients than in the younger group. The results of the study by Bryant and Zarb's (1998), after 4–16 years of follow‐up in two groups consisting of 39 older adults (60–74 years) with 190 implants and 43 younger adults (26–49 years) with 184 implants, also showed that the cumulative implant success rate at the last follow‐up was 92.0% in the older group compared with 86.5% in the younger group. The number of patients older than 75 years with an implant‐supported prosthesis has increased in Switzerland over the past 20 years (Schneider et al., 2017). In the prospective study by Choi et al. (Choi et al., 2023), implant stability was evaluated immediately postoperatively and at 2, 4, and 8 weeks postoperatively in patients in four age groups: <60 years, 61–70 years, 71–80 years, and >80 years. The results showed that patients aged <60 years and 61–70 years had similar implant stability over time. However, unexpectedly, patients aged 71–80 years and those >80 years showed consistent implant stability over time with no loss of stability. In addition, patients >70 years of age demonstrated significantly greater implant stability than younger patients at 2 and 4 weeks after implant placement. Therefore, age does not appear to be a factor of major prognostic significance in dental implant treatment.

Overall, the dental implant scenario is an evolving field where new technologies have been introduced in recent years. For example, the finite element analysis is certainly a valid tool for medical research. The real‐time response, the possibility to perform several analyses in different zones of the system such as the crown, implant, or bone, the accuracy of the models and the results have justified its great popularity among dentists. Nevertheless, the numerical construction of a biological object requires a deep understanding of the instrument otherwise, the validity of the study is lost. Moreover, taking into account the potential error that can occur during surgical treatment, the clinicians are currently considering the use of the sophisticated technique known as guided surgery, that is, an accurate and solid method based on the engineering optimization of the entire workflow. Starting from the scanning of patient's oral cavity and the construction of a 3D model, it provides the employment of surgical guides, realized by specific treatments like stereolithography. In this way, the dental implants are placed more efficiently and precisely that results relevant for a correct implant restoration, minimizing the risk of damage the jawbone.

## 5 CONCLUSIONS

The results of the study, according to the strategic vector identified in the Twisting Zone Adjustment type section, showed that implant surgery is a widespread technique but needs improvement to increase the likelihood of success and reduce the complications that can lead to implant failure. Potential problems of dental implants can be either biological or mechanical in origin, in accordance with the results of our study (SWOT threat matrix). To activate intervention strategies to respond to threats, based on the results of the SWOT matrices, the first approach (opportunity subfactors are the most important requirement) is to aim at the activation of good mechanical performance and good resistance to oral physicochemical agents of dental implants. The material used in dental implants must possess specific physical, mechanical, chemical, and biocompatibility properties, such as mechanical toughness and hardness, as well as resistance to corrosion caused by continuous exposure to external weathering. The AHP results showed among the weaknesses (second most important factor), that pathologies such as bruxism and chronic diseases were considered important factors that may play in favor of dental implant success or failure, followed by poor oral hygiene and smoking. On the other hand, in agreement with the results of our AHP, among the strength categories, one of the predictors of dental implant success is the dentist's adequate competence and experience in the diagnosing, planning, and implementation of implant‐prosthetics rehabilitation. Another finding from the AHP results of our study is that implant treatment in the old population is comparable to that observed in young adults (Bryant & Zarb, 1998; Engfors et al., 2004). In fact, current studies have shown that the old population has a longer life expectancy not only in terms of duration, but especially in terms of increasing the healthy part of life (Aliberti, De Caro, et al., 2022; Aliberti, Funk, et al., 2022; Aliberti et al., 2023). For similar reasons, implant treatment of old patients is increasing worldwide (Choi et al., 2023). According to several authors (Del Amo et al., 2022; Raikar et al., 2017), the overall success rate, after a period of observation, in the older patients was equal or even slightly better than in the younger group.

## AUTHOR CONTRIBUTIONS

**Silvana Mirella Aliberti**: Conceptualization; methodology; software; validation; formal analysis; writing—original draft preparation; review and editing; supervision; project administration. **Mario Capunzo**: Validation; visualization; supervision; project administration. **Marco De Stefano**: Resources; review and editing; visualization; Figures 1 and 2 were made. **Richard H. W. Funk**: Review and editing; visualization. **Thomas Hoffmann**: Review and editing; visualization. All authors have read and agreed to the published version of the manuscript.

## CONFLICT OF INTEREST STATEMENT

The authors declare no conflict of interest.

## Data Availability

The data sets that support the findings of this study are available from the corresponding author upon reasonable request.
